# Of apples and oranges: Lessons learned from the preparation of research protocols for systematic reviews exploring the effectiveness of Specialist Palliative Care

**DOI:** 10.1186/s12904-016-0110-y

**Published:** 2016-04-18

**Authors:** Jan Gaertner, Waldemar Siemens, Barbara A. Daveson, Melinda Smith, Catherine J. Evans, Irene J. Higginson, Gerhild Becker

**Affiliations:** Clinic for Palliative Care, Medical Center - University of Freiburg, Faculty of Medicine, University of Freiburg, Freiburg, Germany; Department of Palliative Care, Policy and Rehabilitation, Cicely Saunders Institute, King’s College London, London, UK

**Keywords:** Palliative care, Systematic review, Clinical trial, Specialist palliative care, Early palliative care

## Abstract

**Background:**

Agreed terminology used in systematic reviews of the effectiveness of specialist palliative care ((S)PC)) is required to ensure consistency and usability and to help guide future similar reviews and the design of clinical trials. During the preparation of protocols for two separate systematic reviews that aimed to assess the effectiveness of SPC, two international research groups collaborated to ensure a high degree of methodological consensus and clarity between reviews. During the collaboration, it became evident that close attention is needed to (i) avoid ambiguity in the definition of advanced illness, (ii) capture the specialist expertise and prerequisites for SPC interventions, and (iii) the multi-professional and multi-dimensional nature of PC. Also, (iv) the exclusion of relevant studies or (v) impracticality of meta-analyses of the obtained data must be avoided.

The aim of this article is to present the core issues of the discussion to help future research groups to easily identify potential pitfalls and methodologic necessities.

**Core issue discussion:**

Core issues that arose from the discussion are presented along the research questions according to the PICO process:

*Population* (P): Authors should refer to existing definitions of PC to ensure that, even if the review aims to investigate specific patients (e.g. cancer patients), it is important to make clear that PC is applicable for all life-limiting diseases and not limited to end-of-life or cancer.

*Intervention* (I): PC is a core responsibility of all disciplines (general PC). In contrast, SPC demands further training and expertise. Therefore, core tenets of SPC interventions are that they are (i) multi-professional and (ii) aim at the multi-dimensional nature of suffering.

*Outcome* (O): The main goal of PC is multi-dimensional (quality of life, suffering or distress). Yet, meta-analysis may be complex to conduct due to the heterogeneity of the multi-dimensional outcomes. Therefore, the assessment of uni-dimensional measures such as pain can also provide clinically relevant information that is easier to obtain.

**Discussion and conclusion:**

Recommendations for future systematic reviews and clinical trials include: (i) Appraise the experience of other research groups who have produced similar systematic reviews or clinical trials.

(ii) Include studies that meet the multi-professional and multi-dimensional nature of PC and the specialization requirements for SPC.

(iii) Thoroughly weigh relevance and practicability of the primary outcome. Multi-dimensional tools such as quality-of-life questionnaires assess the different dimensions of suffering (the true scope of PC), but uni-dimensional measures such as pain are easier to assess in meta-analyses.

## Background and aim of the article

As Specialist Palliative Care (SPC) services develop internationally, there is a growing number of research groups producing evidence-based recommendations or guidelines for such services [1]. During the preparation of protocols for a systematic review [2, 3] that aim to assess the effectiveness of SPC, two separate research groups exchanged and discussed their research protocols to ensure consistency between the reviews and to achieve a high degree of methodological consensus. It became evident, that due to the substantial increase in research activity in the field of PC, the heterogeneity of the trial designs and the choice or absence of core definitions (e.g. for SPC as an intervention), it is crucial and challenging to apply methodological standards that (i) avoid ambiguity in the definition of advanced illness, (ii) capture the specialist expertise and prerequisites for SPC interventions and (iii), the multi-professional and multi-dimensional nature of PC whilst avoiding (iv) the exclusion of relevant studies or (v) impracticality of meta-analyses of the obtained data.

Notably, three major areas of discussion were encountered when delineating the “PICO principle” (population-intervention-comparison-outcome”) [4] for SPC research. These concern the definitions for the included population (P), interventions (I) and the primary outcome (O). In this article, these key issues that arose from intensive discussion between the two research groups are presented.

The aim of this article is to share the methodological communication and decisions of two research groups. Both groups have been working on systematic reviews on specialist palliative inpatient or home care interventions and consisted of PC researchers and clinicians. Senior members of each group were involved in the discussion of national health-policy questions. The groups discussed the most suitable definitions for the included population (P), interventions (I) and the primary outcome (O) for the preparation of systematic review protocols that explore the body of evidence for effectiveness of SPC.

We hope that our publication may facilitate the design of future clinical trials and systematic reviews in the field of palliative care (PC). For each core issue, the different approaches initially used by each research group are reported, followed by the explanation of the consensus reached after the discussion between both research groups.

## Core issue discussion

### P-population

#### Initial

*Group I* chose the following experience-based definition:

*“Patients with an incurable stage of the disease (or a combination of diseases) that is most likely to progress and lead to the patients’ death despite all other disease-modifying or life-prolonging interventions.”*

*Group II* followed pre-existing definitions:

*“Advanced Illness occurs when one or more conditions become serious enough that general health and functioning decline, and treatments begin to lose their impact. This is a process that continues to the end of life”* [5]. Further, group two included patients with *“advanced (Coalition to Transform Advanced Care (CTAC), 2013), life-limiting (Palliative Care Australia 2005), or life-threatening illness (NCP 2013), which is likely to compromise their quality of life (The WHOQOL Group 1995)”* [2].

The group also provided the International Classification of Diseases (ICD) 10 coding for all of the included malignant and non-malignant heart-, lung-, neurological- vascular-, renal-, liver- and infectious diseases to be as specific as possible (malignant: C00-C97; heart- and vascular: I00-I52, I60-69; renal: N17, N18, N28, I12, I13; liver: K70-K77; lung: J06-J18, J20-22; J40-47, J96; neuro: F01, F03, G10, G12.2, G20, G23.1, G35, G90.3, R54; infectious: B20-B24).

#### Consensus and future perspective

##### Refer to existing definitions

Although the definition proposed by group I more specifically focuses on the inevitability of the patient’s death, both groups agreed that it was most useful and applicable to use the agreed international definition of advanced illness as proposed by group II. Both groups noted that it is of importance, that the patient population should not be restricted to patients near the end of life. Rather, SPC should be applicable early in the disease, depending on the patients’ needs. Nevertheless, future studies may both aim at evaluating the effect of SPC early in the disease and on end-of-life care [5].

### I-intervention

#### Initial

*Group I* decided to be as detailed as possible to ensure the specialization and PC expertise of the SPC teams. They modified the definition of SPC that was previously used in the Cochrane review exploring effectiveness of home PC services [6]:

Care primarily aiming at fostering of life and prevent or reduce suffering was provided by:

either (i)

- physicians (one or more) who have received higher specialist training in PC, and
- nursing staff (one or more nurses) who have received higher specialist training, and
- professionals (one or more) attached to the PC team from a medical, psychological, social, theological or other profession allied to health care who have received further training in PC.

The presence of higher specialist education in PC will be assumed adequate, if the authors would describe the professionals as PC “specialists” or “experts” (e.g. “PC physician”). Specialist education in “care of the dying” (or described with synonyms) or specialist training comprising physical, psychosocial or spiritual core aspects of PC will also be considered appropriate. We will also include an intervention as specialist PC if stated as such by the authors.

or (ii)

- health care professionals (e.g. nurses, physicians) working at least 50 % of their time in PC, who have not received higher specialist education but obtained substantial clinical expertise over years and received in-service training for their job, or
- a uni-disciplinary team of health care professionals (e.g. nurses) working at least 50 % of their time in PC, in which one or more members may have received some specialist training.

*Group II* chose a more general definition: *“Inpatient specialist palliative care varies between settings and countries. In order to allow for these differences, inpatient specialist palliative care will include care for patients with an advanced, life-limiting or life-threatening illness that is likely to compromise the patient’s quality of life in some way with or without pre-bereavement care for unpaid caregivers (provided while the patient is alive and in hospital to either the unpaid caregiver alone or together with the patient) (Higginson 2003). The intervention must be aiming to address the primary outcome of this review and/or a secondary outcome. It must also be delivered by a specialist palliative care team or by a “specialist palliative care”, “palliative care” (but not a generalist palliative care member, as defined in Shipman 2008) or “hospice” staff member.”* [2].

#### Consensus and future perspective

##### Specialization, multi-professional- and multi-dimensional care

Both groups agreed that PC as a therapeutic approach (general or basic PC) is a core responsibility of all disciplines. In contrast, SPC demands further training and expertise. Both groups agreed that the definition of group I may be too precise. This decision was based on the experience of the research group with the previously published Cochrane review about palliative home care [6]. Here, it became evident that the great minority of publications or corresponding authors are able to provide such specific information, as has been requested by group I.

Both groups agreed that two core tenets of SPC interventions are that they (i) are multi-professional and (ii) aim at the multi-dimensional nature of suffering. Thus, interventions aiming for example solely at the physical domain (e.g. pain management) or are reduced to a physician visit may be part of PC but not PC itself. Therefore, group I would keep multi-professionalism and the multi-dimensional goal of care as key criteria to recognize the intervention as SPC.

*For future trials*, we strongly advocate that researchers should be as precise as possible in reporting the degree of specialization of their SPC team and other information as demanded in the detailed definition primarily chosen by group I.

### O-outcome

#### Initial

*Group I:* As the primary outcome, group I decided to assess quality of life (QoL). The rationale for doing so was that QoL is (i) the main goal of PC and (ii) multi-dimensional by definition [7]. The latter is of importance to distinguish PC from interventions aimed primarily at single dimensions of QoL, such as pain control (physical domain), depression (psychological domain), family interactions (social domain) or spiritual well-being (spiritual domain). We are aware of the diversity of assessment tools and the difficulty associated with heterogeneity. Yet, we believe that meta-analysis may be possible and could add meaningful insights in some cases. If, in such cases, different QoL measures were identified in the included studies and a meta-analysis for continuous outcomes would be meaningful, we suggest the use of the standardized mean difference. However, absolute changes on the original QoL scales should also be stated (e.g., in the running text or in a separate table) to facilitate the interpretation of the findings. For such meta-analyses, we prefer the use of both the random- and the fixed-effects model, to take into account the heterogeneity of SPC interventions and *small study effects* [8].

*Group II* chose to examine pain intensity as the primary outcome. The rationale for doing so was that the group recognized the diversity of QoL assessment tools and the difficulty that is associated with heterogeneity for meta-analysis.

#### Consensus and future perspective

##### Primary outcome: Multi-dimensional or pragmatic

Both groups agreed that the main goal of PC is multi-dimensional by nature and definition, be it QoL, suffering or distress. Yet, meta-analysis may be complex to conduct due to the heterogeneity of the tools assessing these multi-dimensional outcomes. Therefore, the assessment of uni-dimensional measures such as pain can also provide clinically relevant information that is easier to obtain.

Ideally, a consensus on the most valuable multi-dimensional measures (e.g., PC needs, QoL and distress) needs to be reached to reduce the heterogeneity of outcome measures in future trials. This would extend the evidence base of PC and facilitate meta-analysis [8].

## Conclusion

The application of vague definitions would result in methodologic shortcomings of systematic reviews and therefore weaken evidence-based findings on the effects of SPC. We are aware that research groups might also wish to adhere to their own, rather than to standard (given) definitions. In this case, the agreed upon definitions should be conveyed as precise as possible.

The research groups suggest the following recommendations for researchers developing protocols for systematic reviews or clinical trials in the field of PC:

(i) Critically appraise the experience of other research groups in regards to the difficulties and key issues encountered in the development of previous systematic reviews or clinical trials. For this, personal communication, careful reading of limitation sections or studying published Cochrane protocols will yield valuable information to identify methodological pitfalls and learning points.
(ii) Close attention should be paid to whether the research protocol correctly identifies studies that meet the multi-professional, multi-dimensional nature of PC and specialization requirements for SPC. For this, careful observation of current practice recommendations for example from PC associations such as the European Association for Palliative Care (EAPC) or the Center to Advance Palliative Care (CAPC) is warranted.
(iii) Researchers should thoroughly weigh relevance and practicability of the primary outcome. In our case, QoL is complex and challenging to evaluate due to its multi-dimensional nature and the diversity of the available tools, while outcome measures such as pain intensity are easier to assess but lack the multi-dimensional aspects of PC.
(iv) In any case, the definitions for each PICO question should be reported in detail in the protocol and the resulting publication to ensure maximal transparency.
